# A multidimensional index with SpO_2_ at rest for predicting progressive pulmonary fibrosis

**DOI:** 10.36416/1806-3756/e20250336

**Published:** 2026-09-14

**Authors:** Soraya Abou El Hosn Cordero, Ana Carolina Lima Resende, Eliane Mancuzo, Karin Storrer, Letícia Ferreira Neves Arantes, Fernanda Aguiar, Maria Raquel Soares, Carlos Alberto de Castro Pereira

**Affiliations:** 1. Disciplina de Pneumologia, Departamento de Medicina, Universidade Federal de São Paulo, São Paulo (SP) Brasil.; 2. Disciplina de Pneumologia, Departamento de Medicina, Universidade Federal de Minas Gerais, Belo Horizonte (MG) Brasil.; 3. Disciplina de Pneumologia, Departamento de Medicina, Universidade Federal do Paraná, Curitiba (PR) Brasil.; 4. Disciplina de Pneumologia, Departamento de Medicina, Universidade Federal de Goiás, Goiânia (GO) Brasil.; 5. Hospital Geral Ernesto Simões Filho, Salvador (BA) Brasil.

**Keywords:** Pulmonary fibrosis, Idiopathic pulmonary fibrosis, Lung diseases, interstitial, Mortality

## Abstract

**Objective:**

To develop a multidimensional weighted score for progressive fibrotic interstitial lung disease (fILD) on the basis of mortality risk and compare the score with the progressive pulmonary fibrosis (PPF) guideline and Efficacy and Safety of Nintedanib in Patients with Progressive Fibrosing Interstitial Lung Disease (INBUILD) trial criteria.

**Methods:**

This was a multicenter retrospective cohort study of fILD patients evaluated from January of 2008 to January of 2020. Disease progression was evaluated by changes in dyspnea, HRCT findings, FVC, DL_CO_, and SpO_2_. Associations between the disease progression criteria and survival were analyzed by means of Kaplan-Meier curves. By means of univariate Cox regression, a score was determined on the basis of the relative weights for the progression criteria. The score was derived and tested within the same cohort. We compared the score with the PPF guideline and INBUILD trial criteria.

**Results:**

A total of 380 patients were evaluated. Cox regression was used in order to assign points to the progression criteria: a decrease of < 90% in SpO_2_ at rest (2 points); disease progression on HRCT (2 points); worsening dyspnea (1 point); a decrease ≥ 3% in SpO_2_ at rest (1 point); a decrease of 5-9% in FVC (1 point); and a decrease ≥ 10% in FVC (2 points). The final score was categorized as follows: low risk, 0-2 points (n = 244), median survival = undetermined; intermediate risk, 3-4 points (n = 61), median survival = 81 months (95% CI, 65-96); and high risk, ≥ 5 points (n = 75), median survival = 57 months (95% CI, 38-75; p < 0.001). A total of 136 patients (35.8%) showed disease progression. Kappa concordance was 0.92 (p < 0.001) for the PPF guideline criteria and 0.81 (p < 0.001) for the INBUILD trial criteria.

**Conclusions:**

A multidimensional weighted score allows us to estimate the risk of progressive fILD. When dichotomized, the concordance with the PPF guideline and INBUILD trial criteria is high. External validation is required before routine clinical application.

## INTRODUCTION

Interstitial lung disease (ILD) is a heterogeneous group of conditions characterized by lung inflammation and fibrosis, which can lead to high morbidity, mortality, and health care utilization.^1^

Idiopathic pulmonary fibrosis (IPF) has a poor prognosis and serves as a model for the progressive fibrosing phenotype. However, some patients with other types of fibrotic ILD (fILD) can also develop a progressive phenotype resembling IPF in pathogenesis, clinical behavior, decline in FVC, and survival.^2^ ^-^ ^5^ The term “progressive pulmonary fibrosis” (PPF) has recently been used in order to encompass this group of conditions.^6^ ^,^ ^7^

A decline in FVC is the most frequently used endpoint in studies of IPF.^8^ ^-^ ^11^ However, because there are limitations associated with examining FVC only, it is necessary to identify other useful endpoints.^11^

Dyspnea is the most important factor influencing the quality of life of patients with lung disease; however, quality of life is a subjective factor.^12^ Consequently, researchers have examined associations of increased dyspnea with other adverse outcomes, as well as between changes in HRCT findings and other criteria.^13^ ^,^ ^14^

Patients with fILD frequently develop resting or exertional hypoxemia, both of which have unfavorable outcomes and increased mortality, as does long-term oxygen therapy.^15^ ^,^ ^16^

Hypoxemia is associated with dyspnea, functional impairment, and lower quality of life.^15^ ^,^ ^17^ ^-^ ^20^ Changes in SpO_2_ as measured by oximetry, an indirect measurement of hypoxemia, constitute a simple and widely available test that can be incorporated as a measure of PPF. 

The criteria that are most often used in order to characterize PPF are those suggested by the Efficacy and Safety of Nintedanib in Patients with Progressive Fibrosing Interstitial Lung Disease (INBUILD) trial and the PPF guideline by the American Thoracic Society/European Respiratory Society/Japanese Respiratory Society/*Asociación Latinoamericana de Tórax*.^5^ ^,^ ^6^ However, other outcomes can be considered.^21^

When different risk factors for progression are compared on the basis of their effects on mortality, the impact of each factor varies. Weighting factors assign importance to each variable on the basis of its relevance to the overall assessment. However, no multidimensional weighted score has been developed to assess the risk of ILD progression. 

The objective of the present study was to develop a multidimensional weighted score including changes in SpO_2_ to characterize fibrosis progression on the basis of the risk of mortality, as well as to compare the weighted score with those reported in the INBUILD trial and the PPF guideline. 

## METHODS

This was a multicenter retrospective cohort study including patients with fILD ≥ 18 years of age. Fibrosis was characterized by the presence of reticular opacities with traction bronchiectasis or bronchiolectasis, with or without honeycombing on HRCT. The patients were recruited across six ILD referral centers in Brazil, and they were followed for at least 24 months. 

We identified patients by reviewing their medical records from January of 2008 to January of 2020, with ILD diagnoses made by a multidisciplinary team. Cases of uncertain diagnosis (n = 21) were reviewed by the coordination center at UNIFESP. 

The exclusion criteria were as follows: long-term oxygen therapy; SpO_2_ ≤ 89% at baseline; FVC ≤ 45%; incomplete data; loss to follow-up; exacerbation at first visit; comorbidities such as muscle weakness, congestive heart failure, and chronic pulmonary thromboembolism; and inability to undergo pulmonary function tests. 

The present study was approved by the Research Ethics Committee of the Federal University of São Paulo, located in the city of São Paulo, Brazil (Protocol no. 61172422.1.0000.5505). 

The following variables were recorded at baseline: sex; age; dyspnea as assessed by the modified Medical Research Council scale; history of antigen exposure (for a diagnosis of hypersensitivity pneumonitis [HP]); symptoms indicative of gastroesophageal reflux disease; pulmonary artery systolic pressure as assessed by echocardiography; history of smoking (with never smokers being categorized as nonsmokers and former and current smokers being categorized as smokers); family history of pulmonary fibrosis; autoantibodies; crackles during physical examination; and treatment. 

Ancillary tests included HRCT, pulmonary function tests, SpO_2_ at rest, a panel for autoimmune rheumatic diseases, and, in a proportion of cases, pulmonary biopsies. The main HRCT patterns were classified as fibrotic nonspecific interstitial pneumonia,^22^ definite or probable usual interstitial pneumonia,^6^ and typical or probable fibrotic HP.^23^

We evaluated progression after 6-24 months by measuring changes in dyspnea (classified as worse, stable, or improving on the basis of daily activities), changes in HRCT findings (classified as worse, stable, or improving), declines in pulmonary function variables, and a significant change in SpO_2_ at rest ≥ 3% or a progression to < 90% during follow-up. 

The predicted values for spirometry and DL_CO_ were determined on the basis of reference values for the Brazilian population.^24^ Each center determined its own treatment. 

Descriptive analyses were conducted by calculating means, medians, standard deviations, and frequencies. The distribution of data was evaluated by the Shapiro-Wilk test. The chi-square test and independent sample t-tests were used in order to compare categorical and continuous variables, respectively. 

The thresholds for the disease progression criteria were determined on the basis of previously published values.^5^ ^,^ ^6^ We described the decreases in FVC and DL_CO_ as relative (initial-final/initial) and absolute (initial-final percentage of predicted). We also described decreases in SpO_2_ at rest, worsening of dyspnea, and changes in disease progression on HRCT. 

In all centers, SpO_2_ was measured with patients in a seated position. Because the use of long-term oxygen therapy was an exclusion criterion, all measurements were obtained while patients were breathing room air (FiO_2_ = 21%). Because this was a multicenter retrospective study, different commercially available pulse oximeters were used across the different centers. 

Regarding the cutoff points for SpO_2_ and FVC, a decline ≥ 3% in SpO_2_ was derived from a previous study conducted at our center,^25^ in which the cutoff was determined by using ROC curve analysis with mortality as the outcome in a cohort of 132 patients with IPF. For FVC, the 5% and 10% decline thresholds are well established and widely reported in the literature.^5^ ^,^ ^6^

Exacerbations or hospitalizations were noted but were not used as criteria of disease progression. 

Survival time was analyzed from diagnosis to death from any cause, lung transplantation, or loss to follow-up. Survival times between different cutoff points for disease progression criteria were estimated by the Kaplan-Meier method, and the relative weight for each criterion was calculated by univariate Cox regression analysis. Variables with significant collinearity were excluded. We summarized the results as hazard ratios (HRs) with 95% confidence intervals, and an index was derived from these data. 

The multidimensional index was derived and internally evaluated within the same multicenter cohort. No independent external dataset was available for validation in the present study.

Area under the ROC curve analysis was performed to assess overall performance for each measure and was compared between our score, the INBUILD trial criteria, and the PPF guideline criteria. We dichotomized the score and compared it with the INBUILD trial criteria and the PPF guideline criteria by means of the kappa coefficient. All analyses were performed with the IBM SPSS Statistics software package, version 22 (IBM Corporation, Armonk, NY, USA). The level of significance was set at p < 0.05. 

## RESULTS

A total of 380 patients were included for analysis (supplementary material, Figure S1). The baseline data are shown in Table 1. Most (56.1%) of the patients were female, with a mean age of 61.7 ± 12.4 years. The mean FVC was 71.1 ± 15.8% of the predicted value, and the mean DL_CO_, evaluated in 172 patients, was 56.8 ± 15.8% of the predicted value. 

**Table 1:** Baseline characteristics of patients with fibrotic interstitial lung disease (N = 380).

Characteristic	mean ± SD or n (%)
Age, years	61.7 ± 12.4
Female sex	212 (55.8%)
Autoantibodies	144 (37.9%)
Family history of pulmonary fibrosis	61 (16.1%)
GERD symptoms	204 (53.7%)
Potential hypersensitivity pneumonitis exposure	217 (57.1%)
Current or former smoker	169 (44.5%)
Velcro crackles	264 (69.5%)
PASP, mmHg (n = 124)	35.0 ± 12.3
mMRC dyspnea scale	
0	33 (8.7%)
1	159 (41.8%)
2	143 (37.6%)
3	41 (10.8%)
4	4 (1.1%)
Functional findings	
FVC, % predicted	71.1% ± 15.8
FEV_1_/FVC	0.84 ± 0.09
DL_CO_, % predicted (n = 172)	56.8% ± 15.8
SpO_2_ at rest	95.0% ± 2.1
HRCT pattern	
UIP	84 (22.1%)
Probable UIP	61 (16.1%)
Fibrotic NSIP	158 (41.6%)
Typical or probable fibrotic hypersensitivity pneumonitis	77 (20.3%)
Final ILD diagnosis	
Other	15 (3.9%)
Unclassifiable	38 (10.0%)
Idiopathic pulmonary fibrosis	81 (21.3%)
Hypersensitivity pneumonitis	107 (28.2%)
Connective tissue disease	139 (36.6%)
Systemic sclerosis	62 (16.3%)
Rheumatoid arthritis	29 (7.6%)
Interstitial pneumonia with autoimmune features	20 (5.3%)
Antisynthetase syndrome	11 (2.9%)
Mixed connective tissue disease	9 (2.4%)
Sjögren syndrome	8 (2.1%)

GERD: gastroesophageal reflux disease; PASP: pulmonary artery systolic pressure; mMRC= modified Medical Research Council; UIP: usual interstitial pneumonia; NSIP: nonspecific interstitial pneumonia; and ILD: interstitial lung disease.

A total of 217 patients (57.1%) reported potential HP exposure. However, only 107 had a final diagnosis of fibrotic HP. 

At physical examination, 70% of the patients had velcro crackles. Dyspnea was classified as grade 1 in 41.8% of the patients and grade 2 in 37.6%. 

The main HRCT patterns were fibrotic nonspecific interstitial pneumonia, in 41.6%; usual interstitial pneumonia, in 22.1%; and fibrotic HP, in 20.3%. The most common final diagnosis was connective tissue disease (CTD), in 36.6%, followed by fibrotic HP, in 28.2%, and IPF, in 21.3%. 

A total of 193 patients (50.7%) received glucocorticoid therapy for more than 6 months; 179 (47.1%) received immunosuppressive therapy; and 78 (23.4%) received glucocorticoid and immunosuppressive therapy. During follow-up, 81 patients (21.4%) received antifibrotic therapy. 

Kaplan-Meier curves for mortality based on cutoff values for FVC and DL_CO_ are shown in Figure 1. For FVC, a relative decrease in FVC ≥ 10% in comparison with an absolute decrease was a stronger predictor of reduced survival (Figures 1A and 1B). Regarding DL_CO_, an absolute decrease of 10% and a relative decrease of 15% had similar survival curves (Figures 1C and 1D). 

**Figure 1:**
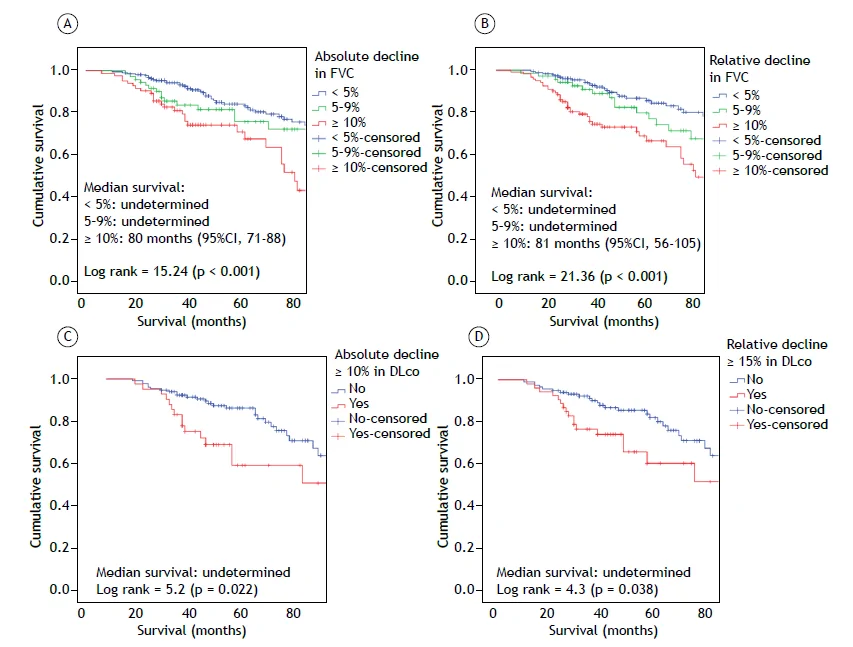
Kaplan-Meier curves for disease progression criteria, by lung function. In A, absolute FVC decline; in B, relative FVC decline; in C, absolute decline ≥ 10% in DL_CO_; and in D, relative decline ≥ 15% in DL_CO_.

A decrease of < 90% in SpO_2_ during follow-up was associated with lower survival, with a median of 45 months (Figure 2A); in addition, a decrease ≥ 3% in SpO_2_ at rest significantly reduced the chance of survival (Figure 2B). 

**Figure 2:**
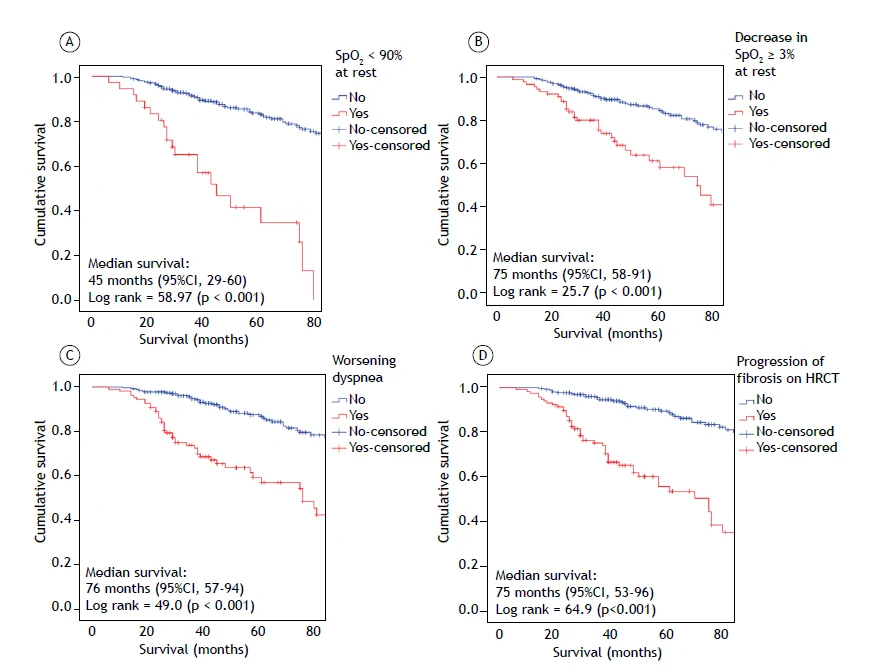
Kaplan-Meier curves for additional disease progression criteria. In A, decrease in SpO_2_ to < 90% at rest during follow-up; In B, decrease in SpO_2_ ≥ 3% at rest; In C, Worsening dyspnea; and in D, progression of fibrosis on HRCT.

The median survival times for patients with worsening dyspnea (Figure 2C) and progression of fibrosis on HRCT (Figure 2D) were similar (76 months and 75 months, respectively). Of the 109 patients with worsening dyspnea, only 8 (7.3%) showed worsening dyspnea as the only disease progression criterion. 

After 6-24 months of follow-up (supplementary material, Table S1), the most common disease progression criterion was a relative decline ≥ 5% in FVC, seen in approximately half of the patients (50.8%). Among nonfunctional variables, fibrosis progression on HRCT (30.8%) and worsening dyspnea (28.7%) were the most common. 

Univariate Cox regression analysis revealed that several factors were associated with a risk of disease progression (Table 2). A decrease in SpO_2_ to < 90% at rest during follow-up was associated with the highest HR (HR = 5.81; 95% CI, 3.49-9.70; p < 0.001). Regarding a relative decline in FVC, Cox regression analysis revealed that a decrease ≥ 10% was the optimal cutoff value for predicting disease progression (HR = 2.54; 95% CI, 1.65-3.90; p < 0.001). 

**Table 2:** Univariate analysis of predictors of disease progression after 6-24 months of follow-up (N = 380).

Criterion for PPF	HR (95% CI)	p
Decrease in SpO_2_ < 90% at rest during follow-up	5.81 (3.49-9.70)	< 0.001
Progression of fibrosis on HRCT	5.10 (3.28-7.93)	< 0.001
Worsening dyspnea	4.11 (2.68-6.32)	< 0.001
Decrease in SpO_2_ ≥ 3% at rest	2.97 (1.91-4.62)	< 0.001
Relative decline ≥ 10% in FVC	2.54 (1.65-3.90)	< 0.001
Relative decline ≥ 5% in FVC	2.36 (1.50-3.71)	< 0.001
Absolute decline ≥ 10% in FVC	2.30 (1.45-3.62)	< 0.001
Absolute decline ≥ 5% in FVC	2.04 (1.33-3.14)	0.001
Absolute decline ≥ 10% in DL_CO_ (n = 172)	2.06 (1.09-3.89)	0.025
Relative decline ≥ 15% in DL_CO_ (n = 172)	1.90 (1.02-3.54)	0.042

PPF: progressive pulmonary fibrosis; and HR: hazard ratio.

A ≥ 3% decrease in SpO_2_ and a decrease in SpO_2_ to < 90% at follow-up were indicative of worse survival (Table 2). As can be seen in Table S1 (supplementary material), the number of patients meeting the criterion of a decrease in SpO_2_ ≥ 3% (n = 89) was higher than that of those meeting the criterion of a decrease in SpO_2_ < 90% (n = 36). 

We used univariate Cox regression to obtain scores, considering the relative weight for risk factors for disease progression as follows: a decrease in SpO_2_ to < 90% at rest during follow-up (2 points); HRCT findings showing progression of fibrosis (2 points); worsening dyspnea (1 point); a decrease ≥ 3% in SpO_2_ at rest (1 point); a relative decline of 5-9% in FVC (1 point); and a relative decline ≥ 10% in FVC (2 points; Table 3). Given that DL_CO_ was not measured in all patients, we created a model that did not include DL_CO_ as a criterion for disease progression. 

**Table 3:** Multidimensional weighted score for progressive fibrotic interstitial lung disease.

Criterion	Score
Decrease in SpO_2_ to < 90% during follow-up	2
Progression of fibrosis on HRCT	2
Worsening dyspnea	1
Decrease in SpO_2_ ≥ 3% at rest	1
Relative decline in FVC%	
< 5%	0
5-9%	1
≥ 10%	2
Risk/score*	Mortality (%)
Low risk (0/1/2)	30 (12.3%)
Intermediate risk (3/4)	20 (32.8%)
High risk (5/6/7/8)	35 (46.7%)

*Χ^2^ = 43.5 (p < 0.001).

Curves with similar survival were merged, and the final score was categorized as follows: low risk (n = 244), 0-2 points, median survival = undetermined; intermediate risk (n = 61), 3-4 points, median survival = 81 months (95% CI, 65-96); and high risk (n = 75), ≥ 5 points, median survival = 57 months (95% CI, 38-75), with p < 0.001. The survival curves for the three groups are shown in Figure 3. 

**Figure 3:**
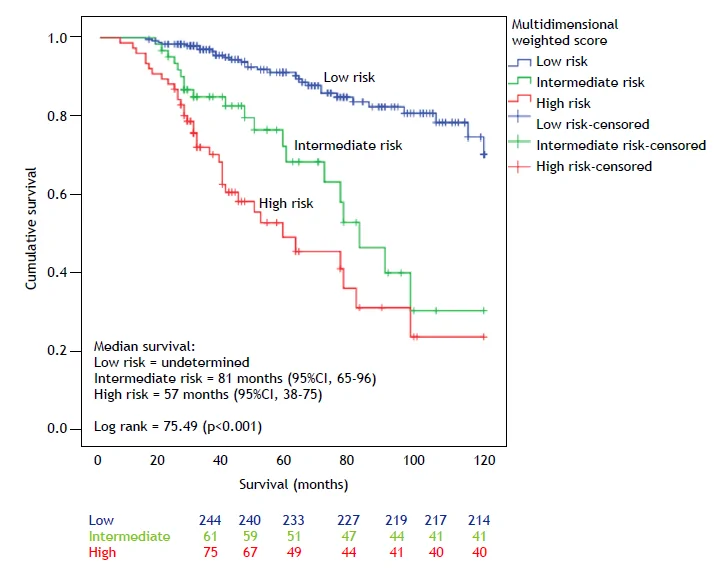
Kaplan-Meier curves for survival according to the final score proposed in the present study, classified as low risk, intermediate risk, or high risk.

At the end of follow-up, a total of 85 patients died, including 30 (12.3%) at low risk, 20 (32.8%) at intermediate risk, and 35 (46.7%) at high risk (Χ^2^ = 43.57; p < 0.001). However, of the 30 low-risk patients who died, 28 experienced exacerbations or were hospitalized (Table 3). 

Patients were dichotomized into low and intermediate/high risk of disease progression, with 136 patients (35.8%) experiencing progression. Fibrotic HP was the disease that was most commonly associated with progression, in 48 patients (35.3%), followed by IPF, in 34 (25%), CTD-associated ILD, in 32 (23.5%), unclassifiable, in 16 (11.8%), and other diseases, in 6 (4.4%; Χ^2^ = 15.8; p = 0.003). In subgroup analyses excluding IPF patients, the log rank was 50.89 (p < 0.001; supplementary material, Figure S2). 

Of the patients who experienced disease progression, 42 (30.9%) received antifibrotic therapy and 94 (69.1%) did not (Χ^2^ = 11.55; p = 0.001). In addition, 101 (74.3%) received glucocorticoid and/or immunosuppressor therapy, whereas 35 (25.7%) did not (Χ^2^ = 0.75; p = 0.38).

The PPF guideline was compared with the score developed in the current study in 172 cases in which DL_CO_ was measured. A total of 113 patients (99.1%) showed no disease progression as assessed by the PPF guideline or the score developed in the present study, and 53 (91.4%) showed disease progression as assessed by the PPF guideline and the present score (kappa = 0.921; p < 0.001). One patient showed disease progression as assessed by the PPF guideline only, whereas another 5 showed disease progression as assessed by our score only. The median survival was 57 months (95% CI, 37-76; log rank = 45.37; p < 0.001; supplementary material, Figure S3). 

The INBUILD trial criteria were also compared with the score developed in the present study (n = 380). A total of 216 patients (88.5%) showed no disease progression as assessed by the INBUILD trial criteria or the present score, and 131 (96.3%) showed disease progression as assessed by the INBUILD trial criteria and the score developed in the present study (kappa = 0.818; p < 0.001). Twenty-eight patients showed disease progression as assessed by the INBUILD trial criteria only, and 5 showed disease progression as assessed by our score only. The median survival was 81 months (95% CI, 64-97; log rank = 43.41; p < 0.001; supplementary material, Figure S4). 

When we compared the overall performance of our score with that of the INBUILD trial and PPF guideline criteria, we found that all had similar ranges. Our score and the PPF guideline criteria showed higher discriminatory ability than did the INBUILD trial criteria, with AUC values of 0.70-0.71 (supplementary material, Figure S5). 

## DISCUSSION

In the present study, we developed a multifactorial scoring system for assessing PPF. We classified the risk of disease progression as low, intermediate, or high. This new score considers different progression criteria based on survival risk and notably replaces DL_CO_ with SpO_2_-an inexpensive and easily evaluable parameter in clinical practice-as a criterion for measuring disease progression. 

When dichotomized into low and intermediate/high risk of disease progression, the proposed score showed high kappa agreement with the INBUILD trial and PPF guideline criteria, indicating that it captures their key aspects, even with the inclusion of patients with IPF. Agreement was stronger with the PPF guideline criteria, as assessed by a higher kappa and good AUC. This is likely due to the fact that both incorporate gas exchange parameters, i.e., DL_CO_ in the PPF guideline and SpO_2_ in our scoring system. Although DL_CO_ is part of current PPF guideline definitions, its limited availability in real-world settings supports the practical advantage of SpO_2_-based models. 

We included patients with IPF for comparative analysis not only because of the well-established characteristics and the natural history of progression as a reference point but also because of the uncertain diagnosis of IPF in those with HRCT patterns suggesting “probable IPF.”^26^ The similarity of findings between IPF and other fILD highlights the need to recognize the progressive phenotype throughout the disease spectrum and questions the strict separation between IPF and PPF. 

When the proposed score was dichotomized into low risk and intermediate/high risk of disease progression, 36% of the patients in our cohort were considered to have PPF. Fibrotic HP was the disease that was most commonly associated with progression, whereas CTD-associated ILD was the least associated with disease progression. 

In a previous review of ten different cohorts of ILD patients, the mean prevalence of progressive disease was 30%.^27^ ^,^ ^28^ In the largest cohort published thus far (N = 2,746), half of the patients met the criteria for PPF, with similar prevalence rates of progression among fibrotic HP and IPF patients.^29^

In our study, a lower proportion of progressive cases was observed among patients treated with antifibrotics. Because antifibrotic therapy can slow disease progression, treated patients might show less physiological decline or radiological worsening, and this might result in lower scores despite similar baseline risk profiles. 

Several criteria have been proposed to characterize progression of ILD.^5^ ^,^ ^6^ ^,^ ^21^ The most widely used criterion involves changes in FVC. The correlation between changes in FVC and mortality in patients with IPF or fibrotic HP has been consistently demonstrated.^11^ ^,^ ^30^

Changes in FVC can be expressed as either a continuous variable or defined on the basis of predefined thresholds. Although continuous change analysis offers increased sensitivity, setting FVC decline thresholds has multiple advantages.^11^ A categorical decline (relative or absolute) ≥ 10% in FVC, as well as marginal declines between 5% and 10% within a six-month period, are associated with an increased risk of death and should therefore be considered clinically relevant changes.^2^

The PPF guideline suggests that an absolute decline > 5% in FVC predicted within 1 year of follow-up constitutes a clinically relevant change, whereas the INBUILD trial suggested that a relative decline of 10% in FVC predicted in the past 24 months constituted a clinically relevant change.^5^ ^,^ ^6^ In our study, a relative decline in FVC (change in the observed percentage divided by the initial value) was more strongly correlated with mortality than was an absolute change in FVC (difference between changes in the predicted value). The same phenomenon was observed in a large study.^31^

Richeldi et al. compared relative and absolute FVC declines in IPF patients and found that a relative decline in FVC captured more patients than did an absolute decline, without sacrificing prognostic accuracy.^10^ This finding is not surprising because a relative decline in FVC is inflated by a lower FVC% in the denominator, thus indicating more advanced disease. In a more recent study of fILD patients, relative measures of lung function decline were found to outperform absolute measures at the same threshold, with higher discriminative ability.^32^

Dyspnea, a multidimensional symptom, was considered progressive when daily activities and routine tasks became more difficult to perform when compared with previously perceived functional capacity. Consistent with our findings, Wijsenbeek et al. reported that worse dyspnea at baseline and over time were associated with a higher risk of disease progression.^33^

SpO_2_ is a parameter that can be used in order to measure hypoxemia. In our study, patients with baseline SpO_2_ ≤ 89% were excluded because they were likely in advanced disease stages, as evidenced by studies showing correlations between disease severity and long-term oxygen therapy when SpO_2_ < 90%.^34^ ^,^ ^35^ By excluding such patients, the score primarily reflects risk stratification among those with mild to moderate disease. 

In our analysis, SpO_2_ < 90% at rest during follow-up was associated with mortality and was a predictor of disease progression. One international cohort study revealed that resting and exertional hypoxemia increased the prognostic value in patients with fILD beyond the Gender-Age-Physiology index, suggesting that SpO_2_ is valuable when measured in combination with age and changes in FVC and DL_CO_.^15^

In a previous study conducted at our center and involving patients with IPF, 45 patients with a decrease in resting SpO_2_ ≥ 3% had shorter survival (27 months; 95% CI, 14-40 months) in comparison with 87 patients without such a decrease (66 months; 95% CI, 44-80 months; p = 0.001).^25^ These results were replicated in the present study. 

Radiological progression can be characterized by several changes; however, such changes were not individualized in this or other studies. In our study, changes in HRCT findings were examined via direct comparisons and were used in order to predict disease prognosis. 

In recent studies, the extent of fibrosis on HRCT has been measured by quantitative analysis, with the most recent studies using deep learning technology.^36^ One study showed that the progression of fibrosis on HRCT was the strongest predictor of subsequent FVC decline,^37^ and a recent model developed by Thillai et al. showed that lower lung volumes were strongly correlated with FVC.^38^

Marinescu et al. found that the extent of honeycombing and traction bronchiectasis was associated with an increased risk of mortality and lung transplantation. The association between radiological findings and guideline-defined patterns was tested, showing that radiological findings superseded the prognostic value of the patterns.^39^ The prognostic factors related to specific variations in the fibrosis score, such as the exact threshold of change that would indicate functional or clinical worsening, remain unclear. 

Our study has several limitations. First, because of the retrospective nature of the study, incomplete data resulted in substantial exclusion of patients from the analysis. Second, only 45% of the patients had DL_CO_ measurements, because of the limited availability in some centers. Third, some studies have demonstrated bias in the measurement of SpO_2_ and race.^40^ However, this was not evaluated in the present study. Fourth, dyspnea worsening and radiological progression were subjectively evaluated; that is, interrater agreement was not investigated, and this may have influenced the classification of disease progression. However, our patients were those that are typically assessed in real-world clinical practice, with the methods being consistent with those employed in other studies, such as the INBUILD trial. Moreover, despite the subjective nature of those criteria, their strong association with mortality was maintained, supporting their relevance and consistency. Finally, an important limitation of the present study is that the proposed index was developed and tested within the same cohort, without external validation in an independent population. Although the internal performance metrics were robust, the absence of external validation limits the generalizability of the model and suggests that the score should be interpreted as preliminary and hypothesis-generating. Prospective external validation is needed to confirm the clinical utility of our multidimensional index for PPF and to assess its role in predicting therapeutic response. A prospective multicenter study for validation of our score is currently in the final stages of planning. 

In conclusion, we developed a multidimensional weighted score without DL_CO_ to evaluate disease progression in patients with fILD. Although our results are hypothesis-generating, they highlight that long-term changes in resting SpO_2_ proved to be valuable in characterizing disease progression in patients with fILD. The present PPF score can be expanded to include biomarkers and other patient-reported outcomes in order to increase its clinical usefulness and predictive accuracy. Nevertheless, our findings represent preliminary evidence and will require prospective, multicenter validation before the score can be applied in clinical practice. Future research should refine scoring systems through longitudinal validation; assess their role in predicting therapeutic response; and support more personalized treatment strategies. 
